# A Medical Causerie

**Published:** 1915-09-25

**Authors:** 


### A MEDICAL CAUSERIE.

The medical aspects of air raids will hardly form a prominent feature in the history of the war, and the majority of the population in the affected areas seem to continue their slumbers undisturbed. In some senses the raids appear to exercise a tonic effect, for many quite mild and peaceful folk are braced up to very vigorous language and to a determination to bring to book the nation which sanctions the deliberate and cold-blooded murder of harmless and innocent children. On the other hand, it is hardly surprising to find that here and there are persons, more especially women, whose nervous systems are upset by the strain and anxiety which explosive bombs cannot fail to cause. Practitioners in the areas exposed to the visits of Zeppelins have thus noticed cases of functional nervous disturbance and of insomnia to be explained in this fashion, while in other instances the symptoms have been those of conspicuous gastro-intestinal disturbance. The latter are probably due in part to exposure to chill, for, good advice to the contrary, people will rush out of their houses when an alarm is given, and very often they are only partially clothed. For those personally injured in these raids the police have well-organised schemes in conjunction with the hospitals and the medical profession, and the arrangements have worked well. Of course there has been no great claim on these for, as Mr. Balfour's letter shows, the number of persons injured has been small. Whether this will continue in the future, time alone can tell, but there is growing confidence that the menace will be successfully met. The threat—and the sequel—of the submarine terror is recalled with satisfaction, and the authorities are believed to be ready to show themselves equal to the occasion. Fortunately, the hospitals have escaped, though this is certainly not due to the goodwill of the invaders.

There appear to be some quarters where the terms "physician" and "surgeon" are regarded as wanting in precision. Perhaps the habit began with the gynæcologists, and it is now showing some very curious results. "Specialist" and "consultant" have been sanctioned, at least in practice, but as they have now been adopted by gentlemen with double-barrelled names who profess to cure obesity and many other evils by "a new method," they may perhaps possess less attraction than they have hitherto enjoyed. "Neurologist" was perhaps the next term which caught on, and "ophthalmologist" was not far behind with various other "ists" in due season. The limit seemed to have arrived when "proctologist" was reached, but on the other side of the Atlantic such names as "pediatrian," "internist," and "gastro-enterologist" have appeared in print, and more may be expected to follow. Another medical point relating to names is an occasional outburst of gratitude in the shape of the conferring on the new baby the name of the medical practitioner in attendance at the youngster's arrival. Hence such baptismal dignities as James Simpson Jones or Lawson Tait Brown are far from being unknown. But what shall be said for the imagination and courage of the father who has conferred upon his son so speaking and significant a title as Occipito Posterior Robinson? Here indeed is a new departure which, if followed, may lead us very far. The advocacy of the study of pre-natal conditions has been pressed with much force, but that any peculiarities in these conditions should be enshrined in the youngster's name can only have occurred to an exceptionally far-seeing mind. If Occipito Posterior Robinson should in later life take the wrong turning it will be useless for him to plead a first offence. His name bewrayeth him and shows he has been wrong from the beginnning; on the other hand, if, as we may hope, he grows up a model of all the virtues, he may be confidently quoted as an example of grace triumphing over Nature.

One pleasing medical outcome of the war is the increased recognition in Great Britain of the medical diplomas of the Universities of the Dominion of Canada. The position has long been anomalous which denied to the graduates of these institutions right to full recognition here, and it was mainly due to mere technical difficulties. Now that these have been overcome we may look forward to the time when a medical qualification granted by a recognised authority in any part of the Empire will be valid for all other parts. In the war the doctors from all parts have worked together and suffered together, and some have died together. Before such experiences the artificial barriers of councils and senates and professors will have to give way. The question of the admission to resident posts in British hospitals of gentlemen of colour and who hold full medical qualifications is not quite so easy. Theoretically the proposal is beyond challenge, and nowhere is there any formal rule against its adoption. But it has not been welcomed in the Metropolitan hospitals, though recent history may quite well lead to some qualification in this practice.

*L'Association Générale des Médecins* of France has had the happy inspiration of establishing a fund for the assistance of the doctors impoverished by the war, and an appeal for support has been issued to all members of the profession in France. It is pointed out that some 14,000 doctors are serving with the Army, and that of these many will return only to find their practices scattered or lost. In addition there is to be considered the position of those who have practised in the invaded parts of the country, and many of whom have lost everything. Whilst some national assistance may be expected this will doubtless be delayed, and in any event there must be many who can count upon no help either from public or private sources. The claim is made that the profession itself should take an active share in providing help for their less fortunate brethren, and the example of the working men who contribute one day's pay each month for a kindred purpose is quoted in support. The amount contributed prior to the issue of the appeal reached a total of more than 45,000 francs.

